# A Review on Recent Treatment Technology for Herbicide Atrazine in Contaminated Environment

**DOI:** 10.3390/ijerph16245129

**Published:** 2019-12-16

**Authors:** Huijun He, Yongpan Liu, Shaohong You, Jie Liu, He Xiao, Zhihong Tu

**Affiliations:** 1 College of Environmental Science and Engineering, Guilin University of Technology, Guilin 541004, China; 2120180313@glut.edu.cn (Y.L.); youshaohong@glut.edu.cn (S.Y.); liujie@glut.edu.cn (J.L.); xiaohe@glut.edu.cn (H.X.); tzh870207@glut.edu.cn (Z.T.); 2 Guangxi Key Laboratory of Theory & Technology for Environmental Pollution Control, Guilin University of Technology, Guilin 541004, China

**Keywords:** atrazine, removal, degradation mechanism, oxidation, plant-microbial remediation

## Abstract

Atrazine is a kind of triazine herbicide that is widely used for weed control due to its good weeding effect and low price. The study of atrazine removal from the environment is of great significance due to the stable structure, difficult degradation, long residence time in environment, and toxicity on the organism and human beings. Therefore, a number of processing technologies are developed and widely employed for atrazine degradation, such as adsorption, photochemical catalysis, biodegradation, etc. In this article, with our previous research work, the progresses of researches about the treatment technology of atrazine are systematically reviewed, which includes the four main aspects of physicochemical, chemical, biological, and material-microbial-integrated aspects. The advantages and disadvantages of various methods are summarized and the degradation mechanisms are also evaluated. Specially, recent advanced technologies, both plant-microbial remediation and the material-microbial-integrated method, have been highlighted on atrazine degradation. Among them, the plant-microbial remediation is based on the combined system of soil-plant-microbes, and the material-microbial-integrated method is based on the synergistic effect of materials and microorganisms. Additionally, future research needs to focus on the excellent removal effect and low environmental impact of functional materials, and the coordination processing of two or more technologies for atrazine removal is also highlighted.

## 1. Introduction

Atrazine (2-chloro-4-ethylamino-6-isopropylamino-1,3,5-triazine) is a triazine herbicide and its molecular formula is C_8_H_14_ClN_5_. It is a white powdery solid and unstable at high temperature. Its melting point is between 173 °C–175 °C, the solubility in water is 33 mg/L at 20 °C and it is easily soluble in organic solvents [1]. Atrazine is often detected in the surface water and groundwater due to its long half-life (30–100 days) [2]. Atrazine can restrain and remove broadleaf weeds and some grass weeds that affect crop growth [3], and also inhibit some perennial weeds. Besides, it is used as a non-selective herbicide on fallow land and non-farmland land. Atrazine is widely used as a herbicide for crops, such as corn, sugar cane, and sorghum, due to its convenience to use, low cost, and excellent weed control efficacy [4]. The annual use of atrazine is 70,000–90,000 tons worldwide [5]. In United States, atrazine has been registered for more than 50 years and it is also the most widely used herbicide in corn cultivation, whose annual dosage is estimated up to 82 million pounds [6,7]. China has been the main producer and user for the past few decades, currently using 1000–1500 tons per year [8], and the area of atrazine-contaminated land exceeds 1.0 × 10^10^ hm^2^ [9]. Although the amount of usage has gradually decreased in recent years, it is still used in corn fields in northern China [10].

The ways of atrazine entering into the environment mainly include precipitation, run-off, and leaching. These behaviors would cause the pollution of soil, surface water and groundwater, and pose a further threat to the ecological environment. Atrazine has been included in the list of endocrine disrupting chemicals (EDCs) by some organizations and countries, such as European Community, Japan, and United States, since it is an environmental estrogen and a potential carcinogen, and it can exist in environment for long time [11]. According to the risk assessment report of US Environmental Protection Agency (EPA), atrazine could have some harmful impact on fish, terrestrial, and aquatic plants, and it might also adversely affect reptiles and amphibians [12]. Atrazine has a significantly toxic effect on animals, and the lethal dose (LD50) for rat and quail is 672 and 5000 mg/kg, respectively [13,14]. Xing et al. [15] found that atrazine induced the methylation of DNA in carp brain and caused the autophagy in the liver. Hayes et al. [16] investigated the growth of frogs in eight regions contaminated by atrazine and found that 92% of frog’s gonads had abnormalities, the egg’s morphology and testis were abnormal, and similar results were obtained in their laboratory studies. In addition, atrazine can also cause harm to human health through respiration, skin contact, etc., which leads to ovarian cancer and breast cancer, and affects the health of human vascular system. For example, the human endocrine system could be damaged with long-term exposure to atrazine [17].

Many countries have set the limitation level for atrazine concentration in the environment, in view of the harm of atrazine to the ecological environment. The maximum concentration of atrazine in primary drinking water in United States is 3.0 μg/L [18]. The European Community also lists atrazine as one of the drinking water testing indicators, and it stipulates that the mass concentration should not exceed 0.1 μg/L [19]. In China, according to the Surface Water Environmental Quality Standard (GB3838-2002), the maximum allowable concentration of atrazine in surface water is 3.0 μg/L, and in the Water Quality Standard for Urban Water Supply (CJ/T 206-2005), the limited concentration of atrazine is 2.0 μg/L. However, the residual amount of atrazine in the environment is still very high due to the extensive usage and the relative stability of the chemical properties. Buser [20] surveyed 18 lakes in Sweden and found that all of these lakes contained atrazine, with the highest concentration reaching to 4.0 μg/L. In Kansas of the United States, the concentration of atrazine in well water was 7.4 μg/L, while the concentration was as high as 25 μg/L in some areas of Minnesota [21]. In the Midwestern of United States, researchers measured the concentration of atrazine in farmland after rainfall, and found that the content was over 300 μg/L [22]. The US Natural Resources Defense Council investigated 153 water systems in United States, and the results showed that 80% of the water samples contained atrazine, and the content of atrazine exceeded the maximum allowable concentration in 65% of the samples [23]. In Europe, the researchers collected 164 groundwater samples from 23 European countries, and the studies showed that atrazine was one of the most common contaminants [24]. Geng et al. [25] studied the organic pollutants in the soil and groundwater in Qian’an and Gongzhuling in Jilin Province, China, and found that the detection rates of atrazine in soil and groundwater were 97% and 89%, respectively.

For the high residue, relative chemical stability, and ecotoxicity of atrazine, many scholars pay high attention to the importance of the studies about how to remove atrazine in the environment and reduce its toxicity from the environment and humans. This review focuses on the treatment technology of atrazine in the environment and summarizes the research progress in this field in recent years from four aspects based on the previous researches of our group: physicochemical, chemical, biological and material-microbial integrated technology. Specially, recent advances regarding material-microbial-integrated technology, based on the physical and chemical properties of materials together with the mineralization of microorganisms, have been highlighted for atrazine degradation. In addition, the paper provides some viewpoints regarding the further researches on atrazine removal.

## 2. Physicochemical Method

The physicochemical method for atrazine treatment is usually based on the adsorption effect. Activated carbon and biochar are the most commonly used adsorbents. Besides, the others, such as bentonite and zeolite, are also frequently used.

### 2.1. Activated Carbon Adsorption

Activated carbon has porous structure and big specific surface area, and these characteristics allow for activated carbon to contain a good adsorption property on various pollutants [26]. Granular activated carbons, powdered activated carbons, and activated carbon fibers are the three most commonly used adsorbents for atrazine removal. Researchers employed activated carbon particles and activated carbon fibers to adsorb atrazine, and the results showed that the adsorption efficiency of atrazine by activated carbon fibers was approximately seven times better than by activated carbon particles [27]. Researchers investigated the combined adsorption property of different materials in order to improve the adsorption effect. Shao et al. [28] removed atrazine with a combination adsorbent of gravity-driven membrane (GDM) and powdered activated carbon, and the removal rate of atrazine by the composite materials under the same conditions was 28.5% times higher than the powdered activated carbon alone. In recent years, the superfine powdered activated carbon (S-PAC) has been developed. Because its particle size was smaller than powdered activated carbon, it had faster adsorption kinetics. As shown in the research of Amaral et al. [29], the removal rate of atrazine by coal-based S-PAC could reach over 90%.

However, in practical applications, activated carbon is limited to widespread application, being generally employed in handling sudden accidents, due to the relatively high cost, secondary pollution, and regeneration difficulty.

### 2.2. Biochar Adsorption

Biochar is produced by pyrolysis and carbonization of biomass materials under the anaerobic or oxygen-deficient conditions [30]. Biochar possesses a certain adsorption performance, so it can be applied in environmental treatments. It can effectively adsorb some organic pollutants, such as polychlorinated biphenyls (PCBs), polycyclic aromatic hydrocarbons (PAHs), and pesticides, such as diuron, carbaryl, acetochlor, and atrazine. Zheng et al. [31] prepared a biochar by pyrolytic decomposition of the mixed bark and wood chips, and found that the biochar could effectively adsorb atrazine under their experimental conditions. Zhang et al. [32] obtained the BC350 and BC700 biochar samples by pyrolytic pig manure at the pyrolysis temperature of 350 °C and 700 °C, respectively. It showed that two materials both had good adsorption capacity for atrazine, and the adsorption effect of BC700 was better than BC350, because of the higher ash content of biochar at higher pyrolysis temperature. Zhao et al. [33] pyrolyzed corn stalk into biochar and used it to remove atrazine; they found that this biochar had strong adsorption capacity for atrazine and its adsorption performance was superior after ammonium dihydrogen phosphate treatment.

When compared with activated carbon, the raw materials for preparing biochar widely exist in environment, and the secondary pollution barely exists during the preparation process. Therefore, biochar is an efficient and environmentally friendly material with broad application prospects. At present, the study on biochar is being in the vigorous development stage. Further research is needed on the selection and proportion of raw materials, optimization of preparation process, and subsequent modification of the adsorption performance.

### 2.3. Other Functional Materials

Other functional materials, such as bentonite, zeolite, and nanoparticle, also have a good adsorption effect on atrazine in addition to activated carbon and biochar. Dutta and Singh [34] studied the adsorption of atrazine by bentonite clay and its modified materials. The results showed that the removal rate of atrazine by bentonite clay was only 9.4%, while the removal efficiencies in bentonites that were modified by stearylkonium (SK) and trioctylmethylammonium (TOMA) were 49% and 72.4%, respectively. Zeolite is also a common adsorbing material, but the untreated zeolite has poor adsorption performance and it needs to be modified to increase the adsorption capacity. Jamil et al. [35] prepared two types of zeolites (X and A) from Egyptian kaolin and studied the adsorption behavior of atrazine; they found zeolite X and zeolite A both had a good adsorption effect on atrazine, and the former, which would adsorb atrazine at lower concentration, was better than the latter in terms of adsorption. In recent years, nanoparticles have also been used for the removal of atrazine with the development of nanotechnology. Some researchers synthesized highly specific molecularly imprinted polymer-nanoparticles (MIP-NPs) via precipitation and mini-emulsion polymerization, and they found that the filtration unit that was packed with MIP-NPs could efficiently remove atrazine at very low concentrations and the MIP-NPs could be regenerated and reused [36]. Li et al. [37] synthesized a magnetic molecularly imprinted polymer on mesoporous silica (mSiO_2_)-coated Fe_3_O_4_ nanoparticles for the adsorption of atrazine; the results showed that the nanomaterials had good adsorption performance for atrazine, and the adsorption capacity was not significantly reduced after five repeated cycles.

As for bentonite and zeolite, they are easy to obtain and consume fewer resources, but their adsorption capacity is low, so improving the adsorption performance is needed for further research. For nanomaterials, most of them possess the excellent adsorption properties. However, the materials are usually expensive, and the preparation process is complicated. Accordingly, the cheap, raw materials and simple preparation process are the future directions.

## 3. Chemical Method

Regarding the chemical methods, organic pollutants are mainly treated by the oxidation-reduction reaction. In this reaction, some chemical substances with strong oxidizing properties (such as hydroxyl radical, sulfate radical, etc.) are generated generally, which can degrade and mineralize atrazine in the environment.

### 3.1. Fenton/Fenton-Like Method

Fenton/Fenton-like methods are to utilize the reaction of H_2_O_2_ and catalysts (such as Fe^2+^) to generate massive hydroxyl radicals (·OH), which have high redox potential and they can oxidize many refractory organic pollutants. They are also employed to remove atrazine in the environment. The typical principle of the reaction is as follows [38]:

(1) Fe^2+^ + H_2_O_2_ → Fe^3+^ + OH^−^ + OH

(2) OH + RH → R + H_2_O

Huang et al. [39] compared various advanced oxidation processes and considered that the Fenton method had the most promising prospects. Chu et al. [40] studied the effect of the dosing method on the atrazine removal by the Fenton reagent. They found that the addition of H_2_O_2_ in a segmented manner was more effective than the typical one-time addition, and the dosage was also saved. Researchers developed various Fenton-like systems based on the Fenton reaction in order to enhance the degradation performance of the Fenton method and reduce the dosage of reagents. For example, Zhang et al. [41] developed a new type of catalyst Ta(O)N, and composed it with H_2_O_2_ and Fe^3+^ to form a Fenton-like system, the system could completely degrade 18 mg/L of atrazine after being irradiated for 60 min. under 500 W xenon lamp. Yang et al. [42] added the functionalized multi-walled carbon nanotubes to the Fenton system, and the degradation rate of atrazine was up to 90% within 30 min. by the new Fenton-like system.

At present, adopting Fenton and Fenton-like methods to purify atrazine in the environment is a common technique, and most of the results show that the Fenton/Fenton-like methods are effective in removing atrazine (Table 1).

Fenton/Fenton-like methods have the advantages of simple operation, rapid reaction, extensive application, and high removal efficiency, but the range of pH values is low in application, and massive sludge is generated during the reaction, so it is limited in practical applications.

### 3.2. Ozone Oxidation Method

As an efficient advanced sewage treatment technology, ozone oxidation is a research hotspot in the field of wastewater treatment in recent years, and it is widely used in the degradation of organic wastewater. The technology can also produce a large amount of hydroxyl radicals (·OH) during the reaction process, and it oxidizes most of refractory organic substances into small molecular substances. The principle of this method is as follows [47]:

(3) O_3_ + OH^−^ → O_2_^−^ + HO_2_

(4) O_3_ + HO_2_ → 2O_2_ + OH

The oxidation activity is not normally very high when only ozone is present. Therefore, improving the oxidation performance of ozone is the major concern for researchers, and adding the catalyst to improve the oxidation performance is one of the important ways (as shown in Table 2). Zhu et al. [48] prepared an ordered mesoporous Fe_3_O_4_ catalyst and used it for the ozonation of atrazine. The results showed that, when compared with the ozone-only oxidation system, the presence of mesoporous Fe_3_O_4_ catalyst greatly improved the oxidation performance of ozone. Yuan et al. [49] synthesized an excellent composite catalyst of graphitic carbon and nitride; the degradation efficiency of atrazine by the catalyst-ozone system was 29.76% times higher than ozonation alone. Some researchers also found that the removal rate of atrazine under ozonation was only 27% at 5 °C, and it was up to 98% under the tourmaline catalytic ozonation system after 10 minutes reaction; the results indicated that tourmaline was effectively used as an ozonation catalyst for degrading atrazine [50]. Saylor et al. [51] studied the effects of the ozone oxidation, electrolysis, and the combined process of the two technologies on the removal of atrazine, and found that the removal efficiency of the combined process was 4.78 times the sum of the two individual processes.

The ozone oxidation method can efficiently oxidize and mineralize refractory organics in the environment, and it does not cause secondary pollution. Additionally, it has the functions of decolorization, disinfection, and deodorization. However, its practical application is limited due to the high cost of ozone generation and the low actual utilization of ozone. Current research should focus on the development of the efficient and economical catalysts to increase the oxidation performance of ozone.

### 3.3. Sulfate Radical (SO_4_^−^·) Oxidation Method

The sulfate radical oxidation method is an emerging advanced oxidation technology in recent years; the oxidation mechanism is that persulfate (PS) or peroxymonosulfate (PMS) is activated by light, heat, or metal ions (M^n+^) to generate sulfate radicals (SO_4_^−^) with super oxidative properties, thereby oxidizing and decomposing the refractory organic pollutants in environment. The activated principle is as follows [57]:

(5) S_2_O_8_^−^ + Heat/UV → SO_4_^−^

(6) M^n+^ + S_2_O_8_^2−^ → M^(n + 1)+^ + SO_4_^2−^ + SO_4_^−^

(7) M^n+^ + HSO_5_^−^ → M^(n + 1)+^ + OH^−^ + SO_4_^−^

There are many activation methods of SO_4_−·for atrazine removal so far (Table 3). Chen et al. [58] removed atrazine by Fe^2+^ activated PS, and found that increasing the concentrations of PS and Fe^2+^ could improve the degradation efficiency of atrazine. They also demonstrated that the degradation efficiency of atrazine was higher under neutral and acidic condition than under the alkaline condition. Wu et al. [59] activated PS with the self-prepared composite of graphene and nanoscale zero-valent iron (nZVI/GR), and studied the performance and mechanism of atrazine degradation under different conditions. The results showed that the removal efficiency of atrazine by this oxidation system was the highest when the mass ratio of nZVI/GR was 5:1. At the same time, they employed the ferrate (Fe(VI))/PMS system to treat atrazine, and it showed that the degradation efficiency of atrazine was significantly enhanced by the Fe(VI)/PMS system as compared with Fe(VI) or PMS alone, and the Fe(VI)/PMS system was effective for atrazine removal over a wide pH range [60].

Sulfate radical oxidation method possesses the strong ability to remove refractory organic pollutants. Because of its superiority, the sulfate radical oxidation method offers the potential for environmental treatment, but activating sulfate radicals to improve its oxidation performance needs further research.

### 3.4. Photocatalytic Method

**Figure 1:**
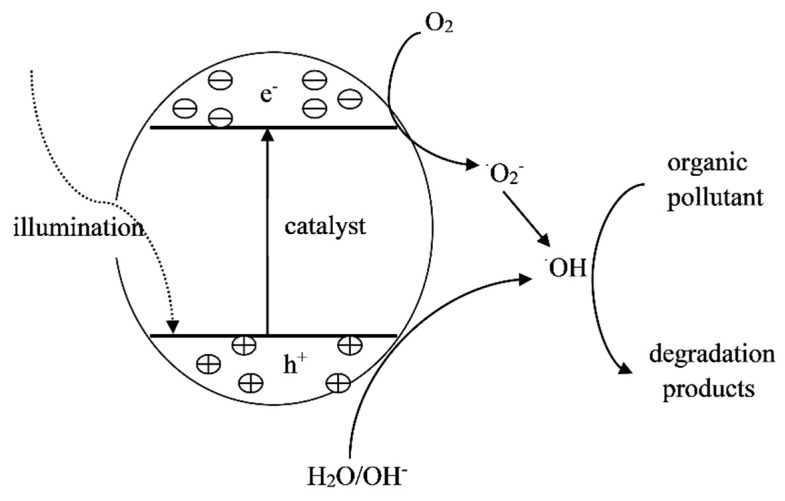
Schematic diagram of photocatalytic degradation.

The photocatalytic method is that added catalysts in reaction system under the irradiation of ultraviolet or visible light produce the strong oxidizing hydroxyl radicals, which oxidize and decompose organic pollutants (Figure 1). Thus, the widely used catalysts include titanium-based, Fe-based, and C-based materials and their oxides (Table 4).

TiO_2_ is one of the commonly used photocatalysts [67]. Some researchers used pure TiO_2_ to degrade atrazine under UV-irradiation and 55% of atrazine was removed after 40 h reaction [68]. It is necessary to improve the catalytic performance by some means, since the photocatalytic efficiency of pure TiO_2_ is generally low [69,70]. Yola et al. [71] employed the reducing agent waste that was produced by industrial process to synthesize a novel nano-TiO_2_ photocatalyst, and successfully applied it to photocatalytic degradation of atrazine. The kinetic studies indicated that the removal of atrazine followed the pseudo-first-order reaction kinetic. Belver et al. [72] prepared a W-TiO_2_/clay photocatalyst by the facile sol-gel method; the composite could effectively remove atrazine under solar light, the degradation efficiency of atrazine reached to 90% after 4.0 h reaction. Besides TiO_2_, researchers also developed other materials with photocatalytic properties. Sudrajat and Sujaridworakun [73] studied the degradation of atrazine by Bi_2_O_3_ nanoparticles under ultraviolet light; the removal efficiency of atrazine was up to 92.1% after 60 minutes irradiation. Sudrajat [74] deposited Cu(I) amorphous nanoclusters on the gC_3_N_4_ surface via ultrasonication successfully, the material possessed a relatively high removal capability for atrazine under visible light, and it observably enhanced the degradation and mineralization of atrazine.

Utilizing the photosensitive characteristics of materials to degrade organic pollutants is a very effective technique. When comparing with the previous adsorption, the Fenton/Fenton-like method and other technologies, it has irreplaceable advantages, such as high degradation performance and reusability of material. However, the quantum yield of many materials is low, and some materials only possess photocatalytic properties under specific illumination. Therefore, in the photocatalytic degradation, it is necessary to study the synthesis of new photocatalysts by selected suitable materials to intensify the catalytic performance in future research.

## 4. Biological Method

Biological treatment technology is widely employed to treat organic pollutants in the environment. It is also a frequently used technique for atrazine degradation, including microbial remediation, phytoremediation, and plant-microbial remediation. Especially in plant-microbial remediation, it a highly effective method that combines phytoremediation and microbial mineralization to degrade organic pollutants.

### 4.1. Microbial Remediation

Prior to the 1980s, atrazine was considered to be one of the most nonbiodegradable organic compounds to microorganisms [11]. However, microorganisms (such as bacteria, fungi) with the biodegradability of atrazine have been gradually identified, separated, and applied to the atrazine removal due to the strong adaptability and the mutability in the polluted environment (Table 5).

The *Pseudomonas* sp. strain ADP was the first to be identified as having the degradation capacity of atrazine [89]. Soon afterwards, other microorganisms with the biodegradability of atrazine were successively isolated and identified. Yang et al. [90] isolated an atrazine-degrading strain TT3 from the soil near the wastewater discharge port of an insecticide factory, and then identified it as *Citricoccus* by 16 S rRNA gene sequencing. The strain could utilize atrazine as the sole nitrogen source to grow, and 50 mg/L of atrazine was completely removed after 66 hours of culture. A strain GZK-1 from a sugarcane field was isolated and then identified as *Arthrobacter* by Getenga et al. [91]; this strain could grow with atrazine as the sole nitrogen source. After 14 days of culture, it was able to mineralize 88% of atrazine at a concentration of 22 mg/L. Researchers studied the synergistic degradation properties of mixed strains in order to improve the degradation ability of microorganisms. Jiang et al. [92] studied the degradation properties of the mixed strains DNS10 and P1. The strain DNS10 was identified as *Arthrobacter* and strain P1 was a phosphorus-dissolving bacterium that could release various organic acids, but lacked the ability of degrade atrazine. The results showed that the mixed strains could remove 99.2% of atrazine, as the atrazine concentration was 100 mg/L after 48 h of reaction, while the single strain DNS10 only degraded 38.6% of atrazine. Yu et al. [93] employed mycelial pellets of *Aspergillus niger* Y3 to immobilize *Arthrobacter* ZXY-2 strain and then used them for treating atrazine. After 10 hours of culture, the mixed organism could completely degrade 57.3 mg/L atrazine, and the organism had good reusability and could be recycled in five batches. In addition, some researchers employed the mixed microorganisms of iron-oxidizing bacteria, coriolus versicolor, and white rot fungi to remove atrazine, and found that the removal rate of atrazine reached 98% by the mixed bacterium [94].

Microbial remediation has such advantages, such as extensive applicable range, relatively simple operation, low running costs, and no secondary pollution. However, at the same time, it has limitations, such as environmental temperature, salinity, pH, nutrient content, toxic substances, and other factors, which will affect the degradation efficiency of microorganisms. Therefore, it is necessary to be search for the microorganisms with better performance and environmental tolerance. In addition, using genetic technology to improve the degradation properties of microorganisms is also receiving increasing attention.

### 4.2. Phytoremediation

Nowadays, many researchers employ phytoremediation technology to deal with the environment that is polluted by atrazine. The removal is mainly attributed to the degradation of some enzymes (such as peroxidase, polyphenol oxidase, and invertase) that are secreted by plant roots, followed by the absorption and transformation of the plants themselves.

Merini et al. [95] remediated soil and water contaminated with atrazine by *Lolium multiflorum*, and found that the removal ability of *Lolium multiflorum* was 20% times higher than natural attenuation. Sanchez et al. [96] investigated the phytoremediation of atrazine with four plants of ryegrass (*Lolium perenne*), tall fescue (*Festuca arundinacea*), barley (*Hordeum vulgare*), and maize (*Zea mays*). The results indicated that all of the plants had the capacity for degrading atrazine, and maize was the plant that most accumulated atrazine among the four plants. Moore et al. [97] degraded atrazine with three plants (*Leersia oryzoides*, *Typha latifolia,* and *Sparganium americanum*), it showed that both *Leersia oryzoides* and *Typha latifolia* significantly reduced the content of atrazine (45% and 35%, respectively), while *Sparganium americanum* did not have the degradability of atrazine. Zhang et al. [98] employed gene-editing technology to prepare a novel engineering rice, which contained a novel metabolic enzyme glycosyltransfearsel (ARGT1) that had the capacity of transforming atrazine. When comparing with common rice, the new rice possessed higher endurance in the atrazine contaminated environment. In recent years, electrokinetic-assisted phytoremediation (EKPR) technology is also employed in the atrazine treatment for enhancing the effect of phytoremediation. The researchers conducted the atrazine removal experiments with electrokinetic-assisted maize phytoremediation. The results indicated that the combined technology significantly enhanced the accumulation of atrazine in plant tissues when comparing with the phytoremediation process alone, and the total accumulation increased by 20–30% [99]. At the same time, they carried out an electrokinetic-assisted ryegrass (*Lolium perenne* L.) phytoremediation test; it showed that the total removal of atrazine by ryegrass increased by 7%, with the help of low voltage gradients [100].

The phytoremediation technology has the advantages of easy operation, environmentally friendly, and it also can beautify the environment. It has been widely used in the treatment of the contaminated environment. However, the processing time of organic pollutants by phytoremediation is relatively long, and some plants have the specificity of the accumulation of pollutants. Besides, the subsequent treatment processes of plants still maintain some difficulties. Furthermore, the post-processing of plants after the phytoremediation is usually incineration after harvesting, but this process might cause secondary pollution [101]. Therefore, the work of screening suitable degraded plants, shortening of the degradation cycle of organic pollutants, and the subsequent treatment of plants urgently require execution.

### 4.3. Plant-Microbial Remediation

Plant-microbial remediation is a combined technology with microorganisms and plants to degrade the pollutants. In the system, the roots of plants provide a favorable place for microbial growth, and the mineralization of organic compounds by microorganisms can provide nutrients for promoting the growth of plants. The mechanism of this technology mainly includes three aspects: plants directly absorb organic pollutants to accumulate or metabolize in the body, the enzymes released by plants promote organic pollutants removal, and the mineralization of microorganisms.

Dong et al. [102] combined arbuscular mycorrhizal fungi *Funnelliformis mosseae* (*F. mosseae*) with *Canna indica* to degrade atrazine in water. They found that the inoculation of *F. mosseae* could alleviate the inhibition of atrazine on the growth and photosynthesis of *Canna indica*, and the highest removal rate of atrazine by *Canna indica* inoculated with *F. mosseae* increased from 68.1% to 95.7% as compared with phytoremediation alone. Bazhanov et al. [103] used the *A. ureafaciens* DnL1-1 to degrade atrazine in combination with wheat or alfalfa; the results showed that the strain could protect plants from the poisoning effect of atrazine. The degradation rates of atrazine by the strain-wheat and strain-alfalfa systems in 30 days reached to 99.8% and 75.6%, respectively. James et al. [104] isolated some *Pseudomonas* strains from the roots of *Acorus calamus*, *Typha latifolia,* and *Phragmites karka*, and employed them for the combined remediation of atrazine. It showed that the combined system of *Acorus calamus* and *Pseudomonas* strains possessed the high removal rate of atrazine, and the combination of microbes-plants could significantly improve the removal of atrazine as compared with the remediation of single plants or microorganisms.

Plant-microbial remediation technology has great research value and broad application prospects, due to the low energy consumption, low operating cost, and the large-scale application for organic pollution control. Therefore, this technology has received extensive attention in the field of bioremediation. Finding synergistic effects of microorganisms and plants and exploring the interaction mechanisms between microorganisms and plants during remediation are the focus of current research.

## 5. Material-Microbial Combined Technology

Various combined technologies have been developed in order to degrade organic pollutants preferably. Utilizing the physicochemical property of materials and the mineralization of microorganisms to prepare new composite biomaterials for treating the polluted environment are starting to get the required attention. The typical synthetic method is the immobilization technology [105]. For this technology, microorganisms and materials are fixed together in a limited space by physical or chemical methods to form a biological material that has both physicochemical and biological effects. The novel biomaterial possesses the advantages of good treatment effect, high microbial concentration, and high flexibility, with respect to fluctuating loading rates [106]. At present, some researchers have begun to develop this material in pollutants treatment (Table 6).

Desitti et al. [112] encapsulated the *Pseudomonas* sp. strain ADP (*P*. ADP) bacteria in core-shell electrospun microtubes, which were prepared a reactor and used for atrazine removal. After 50 days of reaction, the reactor has a stable degradation effect to atrazine (the degradation rate was 83.1 ± 3.9%) without adding an external carbon source. Abigail and Das [113] immobilized *Pichia kudriavzevii* Atz-EN-01 cells on clay brick particles and then encapsulated them in a PVA-SA matrix; it found that the removal rate of atrazine was close to 100% by the new biomaterial. Pannier et al. [114] employed the sol-gel process to immobilize the atrazine-degrading bacterial strain *Pseudomonas* sp. ADP in thin silica layers that were coated onto water-retaining carrier materials. The new material was found to have a removal rate of 94% for 20 mg/L atrazine, and it was able to maintain a high atrazine degradation activity after one year of consecutive batch tests.

The new biomaterials possess excellent performance and good tolerance in the treatment of organic pollutants, which is a new research direction in the field of wastewater treatment. However, it has its own defects. For example, the preparation process of the biomaterial might have adverse effects on microbial activity; immobilized media is easily damaged, which leads to the loss of microorganisms and materials, and so on. Aiming at these issues, developing mild preparation conditions, selecting the suitable entrapment media, and preparing new biomaterials with a low effect of environment and recyclability are the future directions of the technology.

## 6. Conclusions

In the above-mentioned treatment technologies of atrazine, the physicochemical method has the advantages of easy operation and short treatment time. However, the method cannot completely remove organic pollutants, and it is not widely used in practical application. The chemical method has a wide application range due to its effective degradation of organic pollutants. Whereas, when comparing with other treatment technologies, the operating cost of chemical method is relatively high, and hazardous intermediates may be generated during the treatment. Accordingly, it is limited in practical applications. The biological method is relatively inexpensive and little threats on environment, but the processing time is relatively long, so screening for highly efficient strains and plants has the research emphasis in this field. The new biomaterial combines the advantages of materials and microorganisms, and it possesses the excellent removal performance for atrazine, which is a new research direction in environmental treatments. However, its disadvantages are also obvious, and further research is needed.

In view of the unique physicochemical properties of atrazine, it is difficult to achieve ideal removal efficiency by using a single technique to treat atrazine. Therefore, the synergistic treatment of two or more technologies is the research priority in the field of organic pollutant treatment. In addition, the preparation of functional materials with excellent properties and low environmental impact is also the foci of future research.

## Figures and Tables

**Table 1:** The effect of Fenton/Fenton-like method on atrazine removal.

Reaction system	Removal Effect
Fe^2+^/H_2_O_2_	The kinetic constant of atrazine degradation achieved, and the Fenton system could effectively remove atrazine [43].
Steel converter slag (SCS)/H_2_O_2_	The degradation rate of atrazine was 93.7% under the optimal conditions [44].
Fe^3+^/ tannins /H_2_O_2_	Under the optimal conditions, the degradation efficiency of atrazine reached 98% after 30 min. reaction [45].
UV/ S_2_O_8_^2−^/Fe^2+^/H_2_O_2_	The system had obvious synergistic effects and could completely degrade atrazine after 30 min. of reaction [46].

**Table 2:** Degradation of atrazine by catalytic ozonation.

Technical Method	Removal Effect
Zn^0^ immobilized g-C_3_N_4_ catalyzed ozonation	The composite exhibited superior degradation activity with an improvement of 61.2% on atrazine in 1.5 min reaction [52].
Hydroxylamine catalyzed ozonation	Approximately 80% of atrazine was degraded by ozonation in the presence of hydroxylamine, while only 20% of atrazine was degraded by ozonation alone [53].
Iron electrode catalyzed ozonation	When the applied current increased to 20 mA, the removal rate of atrazine increased to 89.8% which the rate was significantly improved compared with ozonation alone [54].
TiO_2_ catalyzed ozonation	Compared with the separate ozonation system, the TiO_2_-ozone system could produce more ·OH, and the degradation rate of atrazine reached 93% after 30 minutes of reaction [55].
Nano-ZnO catalyzed ozonation	The system showed obvious synergistic effect, the degradation efficiency of the system to atrazine was increased by 41.8%, and the degradation reaction was accorded with the pseudo-first-order kinetics [56].

**Table 3:** Different activation methods of SO_4_^−^ for atrazine removal.

Activation Method	Removal Effect
Dithionite activated PS	The system could completely degrade atrazine within 90 min. and the degradation reaction followed the pseudo-first-order kinetics [61].
Copper sulfide (CuS) activated PS	The removal efficiency of atrazine by the system was 91.5% after 40 min. reaction when the concentrations of PS and CuS were 4.0 and 25 mmol/L, respectively [62].
Fe_3_O_4_^−^sepiolite composite activated PS	As the PS concentration of 92 mmol/L, the system could remove 71.6% of atrazine after 60 min. reaction [63].
Ascorbic acid (AA) activated PS	When added AA to the reaction system, the degradation rate of atrazine was increased by 29 times [64].
Fe_3_O_4_^−^hydroxylamine activated PMS	The degradation rate of the system to atrazine was 38 times comparing to the Fe_3_O_4_/PMS system [65].
Graphitic-carbon nitride composites activated PMS	Under the irradiation of xenon lamp, the system could achieve the removal of 78.76% atrazine in 120 min. reaction [66].

**Table 4:** Photocatalytic degradation of atrazine by catalysts.

Photocatalyst	Preparation Method	Removal Effect
Ordered mesoporous graphene–TiO_2_/SiO_2_ composite material	Used a direct sol–gel co-condensation method	The degradation efficiency of atrazine by the composite reached 93.1% after 180 minutes of xenon lamp irradiation [75].
N, F-codoped TiO_2_ nanowires	Synthesized by hydrothermal method using isopropanol as a protective capping agent	The material could effectively degrade atrazine, and the removal rate exceeded 60% after 6.0 h of visible light irradiation [76].
Fe^3+^-TiO_2_	Prepared by a cell gel method	After exposure to UV for 2.0 h, the degradation efficiency of the catalyst to atrazine was as high as 99.5% [77].
N-TiO_2_	A modified sol-gel method was employed to prepare the material	The removal rate of atrazine by the material reached 79% after 2.0 h of visible light irradiation [78].
H_3_PW_12_O_40_/Ag-TiO_2_	Preparation of the nanocomposite by single-step sol-gel-hydrothermal method	Under the xenon lamp, the degradation rate of atrazine by the nanocomposite was 2.4 times faster than TiO_2_ alone, and the degradation reaction followed the pseudo-first reaction kinetics [79].

**Table 5:** Microorganisms with the biodegradability of atrazine.

Strain Name	Strain Source	Strain Category	Removal Effect
ZXY-2	Soil samples near a pesticide factory	*Arthrobacter*	Complete degradation of 100 mg/L atrazine within 15 h [80].
TES6	Corn field	*Arthrobacter*	30 mg/L of atrazine was completely degraded after 3.0 h [81].
HB-6	Industrial wastewater	*Bacillus subtilis*	The degradation rate of 200 mg/L atrazine reached 90% after 24 h [82].
A02	Soil samples	*Pseudomonas*	After 24 h, the degradation rate of 100 mg/L atrazine was 99% [83].
ZXY-1	Soil samples	*Pseudomonas*	100 mg/L atrazine could be completely degraded within 11 h, and the degradation rate was 9.09 mg/(L·h) [84].
CX-T	Industrial soil	*Ensifer*	Complete degradation of 100 mg/L atrazine within 30 h [85].
EGD-AKN5	Sugarcane field	*Pseudomonas*	Degradation efficiency of 100 mg/L atrazine exceeded 80% within 30 h [86].
HB-5	Industrial wastewater	*Arthrobacter*	After 18 h, the removal rate of 100 mg/L atrazine was 100% [87].
*Trametes versicolor*	Wet sawdust	*Coriolus versicolor*	The degradation rate of atrazine in soil reached 96% after 24 weeks [88].

**Table 6:** The properties of new biomaterials for atrazine degradation.

Material-Microbial Composite	Preparation Method	Removal Effect
Fe_3_O_4_-*Saccharomyces cerevisiae* (*S. cerevisiae*)	Nano-Fe_3_O_4_ and *S. cerevisiae* were encapsulated in a sodium alginate-polyvinyl alcohol matrix	The removal rate of 50 mg/L atrazine by the microspheres was 95.53% under the conditions of 28 °C, pH 7.0 and 150 rpm [107].
Fe_3_O_4_-*Penicillium* sp. yz11-22N2	*Penicillium* sp. yz11-22N2 and nano Fe_3_O_4_ were entrapped in polyvinyl alcohol-sodium alginate gel beads	Under the optimal conditions, the new biomaterial had a removal efficiency of 91.2% for 8.0 mg/L atrazine [108].
Fe_3_O_4_-chitosan (CS)- *S. cerevisiae*	*S. cerevisiae* and nano Fe_3_O_4_ linked with CS through epichlorohydrin were encapsulated in calcium alginate	The removal rate of 2.0 mg/L atrazine was 88% at 25 °C and pH 7.0, and the recycled biomaterial still had a good removal capacity [109].
Polyvinyl alcohol-sodium alginate (PVA-SA)-*Leucobacter* sp. JW-1 cells	*Leucobacter* sp. JW-1 cells were immobilized in PVA-SA beads by immobilized microorganism technique	The new material could completely degraded 50 mg/L of atrazine within 2 days [110].
Sodium alginate (SA)- *Arthrobacter* sp. DNS10	*Arthrobacter* sp. DNS10 was immobilized by a SA gel matrix	Under the optimal conditions, the removal rate of 100 mg/L atrazine by the material was 99.67% within 36 h [111].

## References

[1] Martins E.C., de Freitas Melo V., Bohone J.B., Abate G. (2018). Sorption and desorption of atrazine on soils: The effect of different soil fractions. Geoderma.

[2] Taverna M.E., Busatto C.A., Lescano M.R., Nicolau V.V., Zalazar C.S., Meira G.R., Estenoz D.A. (2018). Microparticles based on ionic and organosolv lignins for the controlled release of atrazine. J. Hazard. Mater..

[3] Mac Loughlin C., Canosa I.S., Silveyra G.R., López Greco L.S., Rodríguez E.M. (2016). Effects of atrazine on growth and sex differentiation, in juveniles of the freshwater crayfish *Cherax quadricarinatus*. Ecotoxicol. Environ. Saf..

[4] Tao Q.H., Tang H.X. (2004). Effect of dye compounds on the adsorption of atrazine by natural sediment. Chemosphere.

[5] Zhang C., Qin L., Dou D.C., Li X.N., Ge J., Li J.L. (2018). Atrazine induced oxidative stress and mitochondrial dysfunction in quail (*Coturnix C. coturnix*) kidney via modulating Nrf2 signaling pathway. Chemosphere.

[6] Williams M.M., Boydston R.A., Peachey R.E., Robinson D. (2011). Performance consistency of reduced atrazine use in sweet corn. Field Crop. Res..

[7] Cleary J.A., Tillitt D.E., vom Saal F.S., Nicks D.K., Claunch R.A., Bhandari R.K. (2019). Atrazine induced transgenerational reproductive effects in medaka (*Oryzias latipes*). Environ. Pollut..

[8] Yue L., Ge C.J., Feng D., Yu H.M., Deng H., Fu B.M. (2017). Adsorption–desorption behavior of atrazine on agricultural soils in China. J. Environ. Sci..

[9] Lin Z., Zhen Z., Liang Y.Q., Li J., Yang J.W., Zhong L.Y., Zhao L.R., Li Y.T., Luo C.L., Ren L. (2019). Changes in atrazine speciation and the degradation pathway in red soil during the vermiremediation process. J. Hazard. Mater..

[10] Wang Q.F., Xie S.G. (2012). Isolation and characterization of a high-efficiency soil atrazine-degrading *Arthrobacter* sp. strain. Int. Biodeter. Biodegr..

[11] Fan X.X., Song F.Q. (2014). Bioremediation of atrazine: Recent advances and promises. J. Soil. Sediments.

[12] Bohn T., Cocco E., Gourdol L., Guignard C., Hoffmann L. (2011). Determination of atrazine and degradation products in Luxembourgish drinking water: Origin and fate of potential endocrine-disrupting pesticides. Food Addit. Contam. Part A.

[13] Wujcik E.K., Londoño N.J., Duirk S.E., Monty C.N., Masel R.I. (2013). An acetylcholinesterase-inspired biomimetic toxicity sensor. Chemosphere.

[14] Casa-Resino I.d.l., Valdehita A., Soler F., Navas J.M., Pérez-López M. (2012). Endocrine disruption caused by oral administration of atrazine in European quail (*Coturnix coturnix coturnix*). Comp. Biochem. Physiol. Part C Toxicol. Pharmacol..

[15] Xing H.J., Wang Z.L., Gao X.J., Chen D.C., Wang L.L., Li S., Xu S.W. (2015). Atrazine and chlorpyrifos exposure induces liver autophagic response in common carp. Ecotoxicol. Environ. Saf..

[16] Hayes T.B., Collins A., Lee M., Mendoza M., Noriega N., Stuart A.A., Vonk A. (2002). Hermaphroditic, demasculinized frogs after exposure to the herbicide atrazine at low ecologically relevant doses. Proc. Natl. Acad. Sci. USA.

[17] Mukherjee D., Kar S., Mandal A., Ghosh S., Majumdar S. (2019). Immobilization of tannery industrial sludge in ceramic membrane preparation and hydrophobic surface modification for application in atrazine remediation from water. J. Eur. Ceram. Soc..

[18] Aggelopoulos C.A., Tataraki D., Rassias G. (2018). Degradation of atrazine in soil by dielectric barrier discharge plasma—Potential singlet oxygen mediation. Chem. Eng. J..

[19] Hou X.J., Huang X.P., Ai Z.H., Zhao J.C., Zhang L.Z. (2017). Ascorbic acid induced atrazine degradation. J. Hazard. Mater..

[20] Buser H.R. (1990). Atrazine and other s-triazine herbicides in lakes and in rain in Switzerland. Environ. Sci. Technol..

[21] Mandelbaum R.T., Wackett L.P., Allan D.L. (1993). Mineralization of the s-triazine ring of atrazine by stable bacterial mixed cultures. Appl. Environ. Microb..

[22] Wirbisky-Hershberger S.E., Sanchez O.F., Horzmann K.A., Thanki D., Yuan C., Freeman J.L. (2017). Atrazine exposure decreases the activity of DNMTs, global DNA methylation levels, and *dnmt* expression. Food Chem. Toxicol..

[23] Yılmaz E., Özgür E., Bereli N., Türkmen D., Denizli A. (2017). Plastic antibody based surface plasmon resonance nanosensors for selective atrazine detection. Mater. Sci. Eng. Part C Mater. Biol. Appl..

[24] Loos R., Locoro G., Comero S., Contini S., Schwesig D., Werres F., Balsaa P., Gans O., Weiss S., Blaha L. (2010). Pan-European survey on the occurrence of selected polar organic persistent pollutants in ground water. Water Res..

[25] Geng Y., Ma J., Jia R., Xue L.Q., Tao C.J., Li C.J., Ma X.D., Lin Y. (2013). Impact of Long-Term Atrazine Use on Groundwater Safety in Jilin Province, China. J. Integr. Agric..

[26] Farooq M., Bell A.H., Almustapha M.N., Andresen J.M. (2017). Bio-methane from an-aerobic digestion using activated carbon adsorption. Anaerobe.

[27] Martín-Gullón I., Font R. (2001). Dynamic pesticide removal with activated carbon fibers. Water Res..

[28] Shao S.L., Feng Y.J., Yu H.R., Li J.Y., Li G.B., Liang H. (2017). Presence of an adsorbent cake layer improves the performance of gravity-driven membrane (GDM) filtration system. Water Res..

[29] Amaral P., Partlan E., Li M., Lapolli F., Mefford O.T., Karanfil T., Ladner D.A. (2016). Superfine powdered activated carbon (S-PAC) coatings on microfiltration membranes: Effects of milling time on contaminant removal and flux. Water Res..

[30] Wu S.H., He H.J., Inthapanya X., Yang C.P., Lu L., Zeng G.M., Han Z.F. (2017). Role of biochar on composting of organic wastes and remediation of contaminated soils-a review. Environ. Sci. Pollut. Res..

[31] Zheng W., Guo M.X., Chow T., Bennett D.N., Rajagopalan N. (2010). Sorption properties of greenwaste biochar for two triazine pesticides. J. Hazard. Mater..

[32] Zhang P., Sun H.W., Yu L., Sun T.H. (2013). Adsorption and catalytic hydrolysis of carbaryl and atrazine on pig manure-derived biochars: Impact of structural properties of biochars. J. Hazard. Mater..

[33] Zhao X.C., Ouyang W., Hao F.H., Lin C.Y., Wang F.L., Han S., Geng X.J. (2013). Properties comparison of biochars from corn straw with different pretreatment and sorption behaviour of atrazine. Bioresour. Technol..

[34] Dutta A., Singh N. (2015). Surfactant-modified bentonite clays: Preparation, characterization, and atrazine removal. Environ. Sci. Pollut. Res..

[35] Jamil T.S., Gad-Allah T.A., Ibrahim H.S., Saleh T.S. (2011). Adsorption and isothermal models of atrazine by zeolite prepared from Egyptian kaolin. Solid State Sci..

[36] Gkementzoglou C., Kotrotsiou O., Koronaiou M., Kiparissides C. (2016). Development of a sandwich-type filtration unit packed with MIP nanoparticles for removal of atrazine from water sources. Chem. Eng. J..

[37] Li X., Ma X.G., Huang R.F., Xie X.W., Guo L.H., Zhang M.Y. (2018). Synthesis of a molecularly imprinted polymer on mSiO_2_@Fe_3_O_4_ for the selective adsorption of atrazine. J. Sep. Sci..

[38] Youssef N.A., Shaban S.A., Ibrahim F.A., Mahmoud A.S. (2016). Degradation of methyl orange using Fenton catalytic reaction. Egypt. J. Pet..

[39] Huang C.P., Dong C.D., Tang Z.H. (1993). Advanced chemical oxidation: Its present role and potential future in hazardous waste treatment. Waste Manag..

[40] Chu W., Chan K.H., Kwan C.Y., Choi K.Y. (2007). Degradation of atrazine by modified stepwise-Fenton’s processes. Chemosphere.

[41] Zhang Y.Y., Du Y.X., Liu D.Q., Bian W.J. (2014). The role of dissolved oxygen in the Ta(O)N-driven visible Fenton-like degradation of atrazine. J. Environ. Chem. Eng..

[42] Yang Z.C., Yu A.Q., Shan C., Gao G.D., Pan B.C. (2018). Enhanced Fe (III)-mediated Fenton oxidation of atrazine in the presence of functionalized multi-walled carbon nanotubes. Water Res..

[43] Zhao K., Quan X., Chen S., Yu H.T., Zhang Y.B., Zhao H.M. (2018). Enhanced electro-Fenton performance by fluorine-doped porous carbon for removal of organic pollutants in wastewater. Chem. Eng. J..

[44] Cheng M., Zeng G.M., Huang D.L., Lai C., Xu P., Zhang C., Liu Y., Wan J., Gong X.M., Zhu Y. (2016). Degradation of atrazine by a novel Fenton-like process and assessment the influence on the treated soil. J. Hazard. Mater..

[45] Romero R., Contreras D., Sepúlveda M., Moreno N., Segura C., Melin V. (2020). Assessment of a Fenton reaction driven by insoluble tannins from pine bark in treating an emergent contaminant. J. Hazard. Mater..

[46] Khandarkhaeva M., Batoeva A., Aseev D., Sizykh M., Tsydenova O. (2017). Oxidation of atrazine in aqueous media by solar-enhanced Fenton-like process involving persulfate and ferrous ion. Ecotoxicol. Environ. Saf..

[47] Gong Y.Y., Zhao D.Y. (2017). Effects of oil dispersant on ozone oxidation of phenanthrene and pyrene in marine water. Chemosphere.

[48] Zhu S.M., Dong B.Z., Yu Y.H., Bu L.J., Deng J., Zhou S.Q. (2017). Heterogeneous catalysis of ozone using ordered mesoporous Fe_3_O_4_ for degradation of atrazine. Chem. Eng. J..

[49] Yuan X.J., Xie R.L., Zhang Q., Sun L., Long X.J., Xia D.S. (2019). Oxygen functionalized graphitic carbon nitride as an efficient metal-free ozonation catalyst for atrazine removal: Performance and mechanism. Sep. Purif. Technol..

[50] Wang D., Xu H.D., Ma J., Lu X.H., Qi J.Y., Song S. (2018). Strong promoted catalytic ozonation of atrazine at low temperature using tourmaline as catalyst: Influencing factors, reaction mechanisms and pathways. Chem. Eng. J..

[51] Saylor G.L., Zhao C., Kupferle M.J. (2018). Synergistic enhancement of oxidative degradation of atrazine using combined electrolysis and ozonation. J. Water Process Eng..

[52] Yuan X.J., Qin W.L., Lei X.M., Sun L., Li Q., Li D.Y., Xu H.M., Xia D.S. (2018). Efficient enhancement of ozonation performance via ZVZ immobilized g-C_3_N_4_ towards superior oxidation of micropollutants. Chemosphere.

[53] Yang J.X., Li J., Dong W.Y., Ma J., Cao J., Li T.T., Li J.Y., Gu J., Liu P.X. (2016). Study on enhanced degradation of atrazine by ozonation in the presence of hydroxylamine. J. Hazard. Mater..

[54] Zhou S.Q., Bu L.J., Shi Z., Bi C., Yi Q.H. (2016). A novel advanced oxidation process using iron electrodes and ozone in atrazine degradation: Performance and mechanism. Chem. Eng. J..

[55] Yang Y.X., Cao H.B., Peng P., Bo H.M. (2014). Degradation and transformation of atrazine under catalyzed ozonation process with TiO_2_ as catalyst. J. Hazard. Mater..

[56] Yuan X.J., Yan X., Xu H.M., Li D.Y., Sun L., Cao G., Xia D.S. (2017). Enhanced ozonation degradation of atrazine in the presence of nano-ZnO: Performance, kinetics and effects. J. Environ. Sci..

[57] Rodríguez-Chueca J., Garcia-Cañibano C., Sarro M., Encinas Á., Medana C., Fabbri D., Calza P., Marugán J. (2019). Evaluation of transformation products from chemical oxidation of micropollutants in wastewater by photoassisted generation of sulfate radicals. Chemosphere.

[58] Chen L.W., Hu X.X., Yang Y., Jiang C.L., Bian C., Liu C., Zhang M.Y., Cai T.M. (2018). Degradation of atrazine and structurally related *s*-triazine herbicides in soils by ferrous-activated persulfate: Kinetics, mechanisms and soil-types effects. Chem. Eng. J..

[59] Wu S.H., He H.J., Li X., Yang C.P., Zeng G.M., Wu B., He S.Y., Lu L. (2018). Insights into atrazine degradation by persulfate activation using composite of nanoscale zero-valent iron and graphene: Performances and mechanisms. Chem. Eng. J..

[60] Wu S.H., Li H.R., Li X., He H.J., Yang C.P. (2018). Performances and mechanisms of efficient degradation of atrazine using peroxymonosulfate and ferrate as oxidants. Chem. Eng. J..

[61] Song W., Li J., Fu C.X., Wang Z.Y., Zhang X.L., Yang J.X., Hogland W., Gao L. (2019). Kinetics and pathway of atrazine degradation by a novel method: Persulfate coupled with dithionite. Chem. Eng. J..

[62] Peng J.L., Lu X.H., Jiang X., Zhang Y.H., Chen Q.X., Lai B., Yao G. (2018). Degradation of atrazine by persulfate activation with copper sulfide (CuS): Kinetics study, degradation pathways and mechanism. Chem. Eng. J..

[63] Xu X.M., Chen W.M., Zong S.Y., Ren X., Liu D. (2019). Atrazine degradation using Fe_3_O_4_-sepiolite catalyzed persulfate: Reactivity, mechanism and stability. J. Hazard. Mater..

[64] Hou X.J., Zhan G.M., Huang X.P., Wang N., Ai Z.H., Zhang L.Z. (2019). Persulfate Activation Induced by Ascorbic Acid for Efficient Organic Pollutants Oxidation. Chem. Eng. J..

[65] Li J., Wan Y.J., Li Y.J., Yao G., Lai B. (2019). Surface Fe (III)/Fe (II) cycle promoted the degradation of atrazine by peroxymonosulfate activation in the presence of hydroxylamine. Appl. Catal. Part B Environ..

[66] Dangwang D.J.M., Gong Y., Noumi G.B., Sieliechi J.M., Zhao X., Ma N., Yang M., Tchatchueng J.B. (2019). Peroxymonosulfate improved photocatalytic degradation of atrazine by activated carbon/graphitic carbon nitride composite under visible light irradiation. Chemosphere.

[67] He H.J., Cheng Y., Yang C.P., Zeng G.M., Zhu C.Y., Yan Z. (2017). Influences of anion concentration and valence on dispersion and aggregation of titanium dioxide nanoparticles in aqueous solutions. J. Environ. Sci..

[68] Miranda-García N., Suárez S., Sánchez B., Coronado J.M., Malato S., Maldonado M.I. (2011). Photocatalytic degradation of emerging contaminants in municipal wastewater treatment plant effluents using immobilized TiO_2_ in a solar pilot plant. Appl. Catal. Part B Environ..

[69] He H.J., Wu B., Yang C.P. (2018). Effects of fulvic acids and electrolytes on colloidal stability and photocatalysis of nano-TiO_2_ for atrazine removal. Int. J. Environ. Sci. Technol..

[70] Sun S.W., He H.J., Yang C.P., Cheng Y., Liu Y.P. (2019). Effects of Ca^2+^ and fulvic acids on atrazine degradation by nano-TiO_2_: Performances and mechanisms. Sci. Rep..

[71] Yola M.L., Eren T., Atar N. (2014). A novel efficient photocatalyst based on TiO_2_ nanoparticles involved boron enrichment waste for photocatalytic degradation of atrazine. Chem. Eng. J..

[72] Belver C., Han C., Rodriguez J.J., Dionysiou D.D. (2017). Innovative W-doped titanium dioxide anchored on clay for photocatalytic removal of atrazine. Catal. Today.

[73] Sudrajat H., Sujaridworakun P. (2017). Correlation between particle size of Bi_2_O_3_ nanoparticles and their photocatalytic activity for degradation and mineralization of atrazine. J. Mol. Liq..

[74] Sudrajat H. (2017). Reducing agent-free formation of Cu (I) nanoclusters on gC3N4 for enhanced photocatalysis. J. Alloys Compd..

[75] Li K.X., Huang Y., Yan L.S., Dai Y.H., Xue K.P., Guo H.Q., Huang Z.M., Xiong J.J. (2012). Simulated sunlight photodegradation of aqueous atrazine and rhodamine B catalyzed by the ordered mesoporous graphene–titania/silica composite material. Catal. Commun..

[76] Zhang Y.L., Han C., Nadagouda M.N., Dionysiou D.D. (2015). The fabrication of innovative single crystal N, F-codoped titanium dioxide nanowires with enhanced photocatalytic activity for degradation of atrazine. Appl. Catal. Part B Environ..

[77] Shamsedini N., Dehghani M., Nasseri S., Baghapour M.A. (2017). Photocatalytic degradation of atrazine herbicide with Illuminated Fe^+3^-TiO_2_ Nanoparticles. J. Environ. Health Sci. Eng..

[78] Samsudin E.M., Abd Hamid S.B., Juan J.C., Basirun W.J., Kandjani A.E., Bhargava S.K. (2015). Controlled nitrogen insertion in titanium dioxide for optimal photocatalytic degradation of atrazine. RSC Adv..

[79] Xu L., Zang H.M., Zhang Q., Chen Y., Wei Y.G., Yan J.H., Zhao Y.H. (2013). Photocatalytic degradation of atrazine by H_3_PW_12_O_40_/Ag–TiO_2_: Kinetics, mechanism and degradation pathways. Chem. Eng. J..

[80] Zhao X.Y., Ma F., Feng C.J., Bai S.W., Yang J.X., Wang L. (2017). Complete genome sequence of *Arthrobacter* sp. ZXY-2 associated with effective atrazine degradation and salt adaptation. J. Biotechnol..

[81] El Sebaï T., Devers-Lamrani M., Changey F., Rouard N., Martin-Laurent F. (2011). Evidence of atrazine mineralization in a soil from the Nile Delta: Isolation of *Arthrobacter* sp. TES6, an atrazine-degrading strain. Int. Biodeterior. Biodegrad..

[82] Wang J.H., Zhu L.S., Wang Q., Wang J., Xie H. (2014). Isolation and Characterization of Atrazine Mineralizing *Bacillus subtilis* Strain HB-6. PLoS ONE.

[83] Tonelli Fernandes A.F., Braz V.S., Bauermeister A., Rizzato Paschoal J.A., Lopes N.P., Stehling E.G. (2018). Degradation of atrazine by *Pseudomonas* sp. and *Achromobacter* sp. isolated from Brazilian agricultural soil. Int. Biodeterior. Biodegrad..

[84] Zhao X.Y., Wang L., Ma F., Bai S.W., Yang J.X., Qi S.S. (2017). *Pseudomonas* sp. ZXY-1, a newly isolated and highly efficient atrazine-degrading bacterium, and optimization of biodegradation using response surface methodology. J. Environ. Sci..

[85] Ma L.M., Chen S.S., Yuan J., Yang P.P., Liu Y.P., Stewart K. (2017). Rapid biodegradation of atrazine by *Ensifer* sp. strain and its degradation genes. Int. Biodeterior. Biodegrad..

[86] Bhardwaj P., Sharma A., Sagarkar S., Kapley A. (2015). Mapping atrazine and phenol degradation genes in *Pseudomonas* sp. EGD-AKN5. Biochem. Eng. J..

[87] Wang J.H., Zhu L.S., Liu A.J., Ma T.T., Wang Q., Xie H., Wang J., Jiang T., Zhao R.S. (2011). Isolation and characterization of an *Arthrobacter* sp. strain HB-5 that transforms atrazine. Environ. Geochem. Health.

[88] Bastos A.C., Magan N. (2009). *Trametes versicolor*: Potential for atrazine bioremediation in calcareous clay soil, under low water availability conditions. Int. Biodeterior. Biodegrad..

[89] Mandelbaum R.T., Allan D.L., Wackett L.P. (1995). Isolation and characterization of a *Pseudomonas* sp. that mineralizes the *s*-triazine herbicide atrazine. Appl. Environ. Microb..

[90] Yang X.Y., Wei H.Y., Zhu C.X., Geng B. (2018). Biodegradation of atrazine by the novel *Citricoccus* sp. strain TT3. Ecotoxicol. Environ. Saf..

[91] Getenga Z., Dörfler U., Iwobi A., Schmid M., Schroll R. (2009). Atrazine and terbuthylazine mineralization by an *Arthrobacter* sp. isolated from a sugarcane-cultivated soil in Kenya. Chemosphere.

[92] Jiang Z., Zhang X.Y., Wang Z.Y., Cao B., Deng S.J., Bi M.C., Zhang Y. (2019). Enhanced biodegradation of atrazine by *Arthrobacter* sp. DNS10 during co-culture with a phosphorus solubilizing bacteria: *Enterobacter* sp. P1. Ecotoxicol. Environ. Saf..

[93] Yu T.M., Wang L., Ma F., Yang J.X., Bai S.S., You J.Y. (2019). Self-immobilized biomixture with pellets of *Aspergillus niger* Y3 and *Arthrobacter*. sp. ZXY-2 to remove atrazine in water: A bio-functions integration system. Sci. Total Environ..

[94] Hai F.I., Modin O., Yamamoto K., Fukushi K., Nakajima F., Nghiem L.D. (2012). Pesticide removal by a mixed culture of bacteria and white-rot fungi. J. Taiwan Inst. Chem. Eng..

[95] Merini L.J., Bobillo C., Cuadrado V., Corach D., Giulietti A.M. (2009). Phytoremediation potential of the novel atrazine tolerant *Lolium multiflorum* and studies on the mechanisms involved. Environ. Pollut..

[96] Sánchez V., López-Bellido F.J., Cañizares P., Rodríguez L. (2017). Assessing the phytoremediation potential of crop and grass plants for atrazine-spiked soils. Chemosphere.

[97] Moore M.T., Tyler H.L., Locke M.A. (2013). Aqueous pesticide mitigation efficiency of *Typha latifolia* (L.), *Leersia oryzoides* (L.) Sw., and *Sparganium americanum* Nutt. Chemosphere.

[98] Zhang J.J., Gao S., Xu J.Y., Lu Y.C., Lu F.F., Ma L.Y., Su X.N., Yang H. (2017). Degrading and Phytoextracting Atrazine Residues in Rice (*Oryza sativa*) and Growth Media Intensified by a Phase II Mechanism Modulator. Environ. Sci. Technol..

[99] Sánchez V., López-Bellido F.J., Rodrigo M.A., Rodríguez L. (2019). Electrokinetic-assisted phytoremediation of atrazine: Differences between electrode and interelectrode soil sections. Sep. Purif. Technol..

[100] Sánchez V., López-Bellido J., Rodrigo M.A., Rodríguez L. (2019). Enhancing the removal of atrazine from soils by electrokinetic-assisted phytoremediation using ryegrass (*Lolium perenne* L.). Chemosphere.

[101] Wu L.H., Zhong D.X., Du Y.Z., Lu S.Y., Fu D.Q., Li Z., Li X.D., Chi Y., Luo Y.M., Yan J.H. (2013). Emission and Control Characteristics for Incineration of *Sedum Plumbizincicola* Biomass in a Laboratory-Scale Entrained Flow Tube Furnace. Int. J. Phytoremediat..

[102] Dong J., Wang L., Ma F., Yang J.X., Qi S.S., Zhao T. (2016). The effect of *Funnelliformis mosseae* inoculation on the phytoremediation of atrazine by the aquatic plant *Canna indica* L. var. flava Roxb. RSC Adv..

[103] Bazhanov D.P., Yang K., Li H.M., Li C.Y., Li J.S., Chen X.F., Yang H.T. (2017). Colonization of plant roots and enhanced atrazine degradation by a strain of *Arthrobacter ureafaciens*. Appl. Microbiol. Biotechnol..

[104] James A., Singh D.K., Khankhane P.J. (2018). Enhanced atrazine removal by hydrophyte-bacterium associations and in vitro screening of the isolates for their plant growth-promoting potential. Int. J. Phytoremediat..

[105] Gao Q.T., Wong Y.S., Tam N.F.Y. (2011). Removal and biodegradation of nonylphenol by immobilized *Chlorella vulgaris*. Bioresour. Technol..

[106] Shin D.C., Kim J.S., Park C.H. (2019). Study on physical and chemical characteristics of microorganism immobilized media for advanced wastewater treatment. J. Water Process Eng..

[107] Wu X., He H.J., Yang W.L., Yu J.P., Yang C.P. (2018). Efficient removal of atrazine from aqueous solutions using magnetic *Saccharomyces cerevisiae* bionanomaterial. Appl. Microbiol. Biotechnol..

[108] Yu J.P., He H.J., Yang W.L., Yang C.P., Zeng G.M., Wu X. (2018). Magnetic bionanoparticles of *Penicillium* sp. yz11-22N2 doped with Fe_3_O_4_ and encapsulated within PVA-SA gel beads for atrazine removal. Bioresour. Technol..

[109] Zhu C.Y., Yang W.L., He H.J., Yang C.P., Yu J.P., Wu X., Zeng G.M., Tarre S., Green M. (2018). Preparation, performances and mechanisms of magnetic *Saccharomyces cerevisiae* bionanocomposites for atrazine removal. Chemosphere.

[110] Liu J.W., Pan D.D., Wu X.W., Chen H.Y., Cao H.Q., Li Q.X., Hua R.M. (2018). Enhanced degradation of prometryn and other *s*-triazine herbicides in pure cultures and wastewater by polyvinyl alcohol-sodium alginate immobilized *Leucobacter* sp. JW-1. Sci. Total Environ..

[111] Ying Z., Zhang Q.Y., Chao N., Ge S.J., Zhao J., Miao H., Bo C. (2015). Biodegradation of atrazine by free and immobilized cells of *Arthrobacter* sp. strain DNS10. Environ. Eng. Manag. J..

[112] Desitti C., Beliavski M., Tarre S., Avrahami R., Zussman E., Green M. (2017). Durable electrospun microtubes for encapsulation of bacteria in atrazine bioremediation. J. Water Process Eng..

[113] Abigail M E.A., Das N. (2015). Removal of atrazine from aqueous environment using immobilized *Pichia kudriavzevii* Atz-EN-01 by two different methods. Int. Biodeterior. Biodegrad..

[114] Pannier A., Lehrer T., Vogel M., Soltmann U., Bottcher H., Tarre S., Green M., Raff J., Pollmann K. (2014). Long-term activity of biohybrid coatings of atrazine-degrading bacteria *Pseudomonas* sp. ADP. RSC Adv..

